# Unhealthy Lifestyle Contributes to Negative Mental Health and Poor Quality of Life in Young University Students

**DOI:** 10.3390/healthcare12222213

**Published:** 2024-11-06

**Authors:** Felipe Caamaño-Navarrete, Esteban Saavedra-Vallejos, Iris Paola Guzmán-Guzmán, Carlos Arriagada-Hernández, Gerardo Fuentes-Vilugrón, Lorena Jara-Tomckowiack, Roberto Lagos-Hernández, Paola Fuentes-Merino, Cristian Alvarez, Pedro Delgado-Floody

**Affiliations:** 1 Physical Education Career, Faculty of Education, Universidad Autónoma de Chile, Temuco 4780000, Chile; felipe.caamano@uautonoma.cl (F.C.-N.); carlos.arriagada@uautonoma.cl (C.A.-H.); roberto.lagos@uautonoma.cl (R.L.-H.); paola.fuentes@uautonoma.cl (P.F.-M.); 2 School of Physical Education, Faculty of Education, Universidad Santo Tomás, Santiago 8370003, Chile; esaavedra6@santotomas.cl; 3 Faculty of Chemical-Biological Sciences, Universidad Autónoma de Guerrero, Chilpancingo 39000, Mexico; pao_nkiller@yahoo.com.mx; 4 Collaborative Research Group for School Development (GICDE), Temuco 4780000, Chile; gerardo.fuentes@uautonoma.cl; 5 Faculty of Education, Universidad Autónoma de Chile, Temuco 4780000, Chile; 6 Faculty of Education, Universidad Católica de Temuco, Temuco 4780000, Chile; lorenajarat@gmail.com; 7 Exercise and Rehabilitation Sciences Institute, School of Physical Therapy, Faculty of Rehabilitation Sciences, Universidad Andres Bello, Santiago 7591538, Chile; cristian.alvarez@unab.cl; 8 Department of Physical Education, Sport and Recreation, Universidad de La Frontera, Temuco 4811230, Chile

**Keywords:** mental health, quality of life, lifestyle, physical activity, screen time, students

## Abstract

**Background:** A negative lifestyle is reported to be related to poor mental health and quality of life (QOL). However, there is little information on this in university students. The objective of the present study was to investigate the association between mental health (i.e., anxiety, depression symptoms and stress), QOL, SWLS and lifestyle parameters (i.e., PA, sleep duration, ST and food habits) among Chilean university students and then to determine the differences in mental health, QOL, SWLS and lifestyle parameters according to gender. **Methods:** This cross-sectional study included a total of 211 university students (128 females and 83 males) aged 18–28 years. Mental health, QOL and lifestyle were measured through validated questionnaires. **Results:** Bad food habits (lowest score in the food survey) were linked to anxiety (2.3 [0.22–4.36], *p* = 0.03), depressive symptoms (3.75 [1.54–5.9], *p* = 0.001) and stress (2.24 [0.31–4.17], *p* = 0.023). Furthermore, <6 h of sleep was related to poorer mental health (13.5 [7.6–19.5], *p* = 0.001), anxiety (4.2 [2.0–6.4], *p* < 0.001), depressive symptoms (5.5 [3.2–7.9], *p* < 0.001) and stress (3.8 [1.8–5.9], *p* < 0.001). In addition, ≥4 h of ST was linked positively to negative mental health (8.3 [2.86–13.7], *p* = 0.003), depressive symptoms (3.45 [1.47–5.4], *p* = 0.001) and anxiety (3.2 [1.05–5.4], *p* = 0.004). Non-physical activity was related to the scores for anxiety (2.6 [0.20–5.0], *p* = 0.030), depression (2.7 [0.009–5.3], *p* = 0.049) and stress (2.4 [0.12–4.7], *p* = 0.039). **Conclusions:** this study showed that unhealthy lifestyle factors (i.e., insufficient sleep, lack of PA and prolonged ST) were strongly associated with poorer mental health and QOL in university students. These findings highlight the importance of addressing these aspects of lifestyle in intervention and health promotion programs aimed at young university students in order to improve their mental health and overall QOL.

## 1. Introduction

Mental health in university students and young subjects is a public health challenge [1]. It is estimated that around one billion people worldwide suffer from a mental disorder, with 50% of cases starting before the age of 14 years [2]. In addition, mental health and its components are a dimension of growing interest for universities [3]. In this context, mental health is an integral part of health and well-being [4]. Likewise, quality of life (QOL) is defined by the World Health Organization (WHO) as individuals’ perceptions of their position in life in the context of the culture and value systems in which they live and in relation to their goals, expectations, standards and concerns [5], and is considered an important measure of people’s daily functioning [6]. Both mental health and QOL are dimensions of relevance to university students [7].

Previously, it has been indicated that attending university can generate different stressful situations [8]. Complementarily to the above, the transition to higher education can be a challenging time for students, as they undergo a series of crises that can impact their psychosocial and physical development [9]. This can lead to the development of mental health disorders, which is a serious global public health concern [10]. When students begin university, they are in a period of neurological sensitivity and undergoing significant adaptive processes [11]. With the start of a period of family relocation and increased personal autonomy [12], there is a significant rise in intellectual activity and social bonding. Moreover, evidence has shown that this period produces a relevant deterioration in mental health, and it is suggested that almost a third of students worldwide manifest this condition [13], characterized by the appearance of depressive disorders [14], generalized anxiety, panic attacks, increased alcohol and substance use [15] or the presence of stress conditions. In Chile, an investigation among university students reported a high prevalence of anxiety, depressive symptoms and stress [16]. Another study conducted in Chilean university students showed a high prevalence of mental health problems [17]. It has also been indicated that anxiety and depression are the most common mental health problems in Chilean university students [16].

Unhealthy lifestyle has become a topic of great interest due to its consequences on the public health of various countries [18]. Moreover, university students form part of the population most at risk of developing unhealthy lifestyle habits [19]. Complementarily to the above, the university stage has been associated with unhealthy lifestyle choices (i.e., physical inactivity, sedentary behavior, unhealthy food habits, smoking and alcohol consumption) [20,21]. In this context, a longitudinal study among Chilean university students showed that after spending a year at university, the participants had decreased physical activity (PA) and worsened eating habits [22]. Based on previously reported findings, strategies promoting healthy lifestyles may have a beneficial impact for university students [23].

University students’ lifestyles have commonly been investigated in the context of health and it has been shown that lifestyle factors are intertwined with mental health components [23]. In this context, the evidence has shown a positive link between a healthy lifestyle and better levels of mental health [24]. Furthermore, studies have reported a strong association between negative lifestyle and poor mental and psychosocial well-being [25,26]. In particular, poor diet quality has been found to have a negative relationship with mental health in university students [27]. Moreover, research indicates that mental health can impact academic success, social functioning, and university dropout rates. However, there are also protective factors, such as PA [28], which has been shown to have benefits for university students. Likewise, it has been indicated that good sleep quality may improve mental health [29]. In contrast, low levels of PA have been linked to poorer academic performance and increased anxiety symptoms [30]. However, research has demonstrated that engaging in regular PA can reduce perceived stress and improve psychological well-being, which has been found to be positively associated with academic success [31,32]. In this sense, the Canadian 24 h movement guidelines recommend that people have less than two hours per day of recreational screen time (ST) [33]. Also, it has been indicated that ST is one of the most important components of sedentary time in university students [34]. Screen time (ST) also represents a predictor of mental health factors in university students [35]. In this sense, an increase in ST has been shown to affect mental health and health-related quality of life in youth [36].

In addition, previous data regarding young people have indicated that an unhealthy lifestyle (i.e., low PA and poor sleep quality) is linked with poorer QOL [37]. Moreover, a previous study conducted in university students indicated that a healthy lifestyle was positively related to satisfaction with life [38].

In view of this background, the present study aimed to investigate the association between mental health (i.e., anxiety, depression symptoms and stress), QOL, SWLS and lifestyle parameters (i.e., PA, sleep duration, ST and food habits) among Chilean university students and then to determine the differences in mental health, QOL, SWLS and lifestyle parameters according to gender.

In relation to the above, the scientific novelty of this study lies in its focus on the Chilean population, focused specifically on Chilean university students, which facilitates understanding the particularities and needs of this specific population. Furthermore, being a specific study, it allows for decision making at the higher education level adapted to local needs.

## 2. Materials and Methods

### 2.1. Participants

This cross-sectional study included a total of 211 first- and second-year university students (128 females and 83 males) aged 18–28 years. The calculation of the sample was carried out from 486 university students from 3 majors of the Faculty of Education with a confidence level of 95% and statistical power at 80%; the selected number was 216 participants, after excluding 43 subjects for not meeting the inclusion criteria or for other reasons, and the sample analyzed was n = 211. The sample was intentional and non-probabilistic.

The inclusion criteria for the participants involved giving informed consent and being university students of the faculty of education. The exclusion criteria were as follows: any medical contraindications that would hinder their performance in the assessments; not fully completing the information collection instruments; not being present at the time of the assessments and surveys with missing data. The project complied with the Declaration of Helsinki (2013) and was approved by the Ethics Committee of the Universidad Autónoma de Chile, Chile (No. CEC 18-23 Act). All the questionnaires were filled out individually in the presence of previously trained research assistants (to resolve any doubts that might arise) and on university premises. In addition, the questionnaires were administered during the academic year and in classes held in the morning.

### 2.2. Main Outcomes

#### 2.2.1. Lifestyle

PA was evaluated using a short version of the International Physical Activity Questionnaire (IPAQ) [36]. The IPAQ consists of seven questions on frequency, duration and intensity (moderate and intense) of PA over the last seven days. This questionnaire was validated previously in Chilean subjects [39] and used in university students [40]. ST and sleep duration were evaluated using the following questions: “How many hours a week do you watch videos?”; “How many hours a week do you play video or computer games?”; and “How many hours of sleep do you usually get per day and/or night?”. These questions were used in previous investigations [41,42].

To determine eating habits, participants completed a questionnaire that had been previously used in various studies with Chilean university students [43,44,45]. The questionnaire comprises 15 dichotomous questions (yes/no) on eating habits (frequency of consumption of certain foods, adequate food consumption and behaviors related to healthy food habits), and the scores were categorized as follows: ≥13, healthy food habits; 10–12, on the right track but could improve; 7–9 points, unhealthy food habits; ≤6 points, very unhealthy food habits [46].

#### 2.2.2. Mental Health

The abbreviated version of the Depression Anxiety Stress Scale (DASS-21) was used to determine the levels of anxiety, depressive symptoms and stress. This self-report scale was created to determine the presence and intensity of emotional states or symptoms of depression, anxiety and stress [47,48]. The questionnaire is composed of 21 questions (divided into three subscales) with scores of 0–3 according to the degree of intensity with respect to last week (0, “It doesn’t describe anything that happened to me or that I felt during the week”; 3, “Yes, this happened to me a lot or most of the time”). This instrument has the advantage of being a self-report scale that is brief and easy to administer, answer and interpret [49]. The DASS-21 has been used and validated in Chilean university students [49,50] and has presented adequate psychometric properties in previous validation studies [49]. The following cut-off points were used for the interpretation of the DAAS-21: Depression: 5–6, mild depression; 7–10, moderate depression; 11–13, severe depression; 14 or more, extremely severe depression. Anxiety: 4, mild anxieties; 5–7, moderate anxiety; 8–9, severe anxiety; 10 or more, extremely severe anxiety. Stress: 8–9, mild stress; 10–12, moderate stress; 13–16, severe stress; 17 or more, extremely severe stress.

The WHOQOL-BREF scale was used to determine the quality of life. This is a 26-item instrument (divided into four subscales) scored on a five-point Likert scale and the participants respond to questions on the previous two weeks. The domains evaluated by the instrument are physical and psychological health, social relationships and the environment. Higher scores indicate better QOL. This questionnaire has been validated in university students [51,52] and previously used in Chilean adult subjects [53].

The Satisfaction with Life Scale (SWLS) was used to assess satisfaction with life and consists of five items that evaluate global cognitive judgements about one’s own life: “In most ways my life is close to my ideal”; “The conditions of my life are excellent”; “I am satisfied with my life”; “So far I have gotten the important things I want in life”; and “If I could live my life over, I would change almost nothing”. The instrument comprises five questions scored on a five-point Likert scale (1, disagree completely; 5, agree completely) [54], with higher scores linked to better SWLS. This scale was previously used and validated in Chilean university students [55].

### 2.3. Statistical Analysis

Statistical analysis was performed using STATA Version 15. The Kolmogorov–Smirnov test and Levene’s test were used for normally distributed data and for homogeneity of variance, respectively. Numerical variables were analyzed using the Wilcoxon rank-sum test to determine if the medians of the quantitative variables related to negative mental health, lifestyle and quality of life differ between men and women. Statistical significance between qualitative variables was examined with the Chi-square test to investigating the probability of individuals being in specific categories in this study. Associations between study variables were evaluated using multiple lineal and logistic regression; the beta coefficient was determined to assess the change in scores of mental health and quality life related to each unit increase in lifestyle variables; and the odds ratio determined the increment in the probability of presenting the risk category for anxiety, depressive symptoms and relationship stress in the category of poor lifestyle. A *p* value of <0.05 indicates statistical significance.

## 3. Results

The scores related to the DASS-21 were significantly higher in women. When categorizing the severity of negative mental health conditions according to gender, it was found that women presented a higher proportion of severe and extreme anxiety, whereas depressive symptoms and stress were similarly distributed between men and women (Table 1). In relation to QOL, men presented better physical health compared to women, and although the SWLS score was similar between men and women, women presented a higher frequency of categories of dissatisfaction with life.

Table 2 shows the distribution of lifestyle parameters, among which diet, sleep, PA and ST were evaluated. Women presented mostly poor diet quality, whereas men presented more ST but also more PA. The number of hours of sleep was similar between men and women (Table 2).

In a model adjusted by age and gender, bad food habits was associated with anxiety (2.3 [0.22–4.36], *p* = 0.03), depressive symptoms (3.75 [1.54–5.9], *p* = 0.001) and stress (2.24 [0.31–4.17], *p* = 0.023) and inversely with QOL (−12.0 [−17.2 to −6.8], *p* < 0.001) and SWLS (−3.77 [−5.5 to −2.06], *p* < 0.001). Furthermore, <6 h of sleep was positively associated with poorer mental health 13.5 [7.6–19.5], *p* = 0.001), anxiety (4.2 [2.0–6.4], *p* < 0.001), depressive symptoms (5.5 [3.2–7.9], *p* < 0.001) and stress (3.8 [1.8–5.9] *p* < 0.001) and inversely with QOL (−13.8 [−19.4 to −8.2], *p* < 0.001) and SWLS (−4.27 [−6.1 to −2.4], *p* < 0.001). Similarly, ≥4 h of ST was linked positively to negative mental health (8.3 [2.86–13.7], *p* = 0.003), depressive symptoms (3.45 [1.47–5.4], *p* = 0.001) and anxiety (3.2 [1.05–5.4], *p* = 0.004) and inversely with SWLS (−2.15 [−3.86 to −0.43], *p* = 0.015). Finally, non-physical activity was related to the scores for anxiety (2.6 [0.20–5.0], *p* = 0.030), depression (2.7 [0.009–5.3], *p* = 0.049) and stress (2.4 [0.12–4.7], *p* = 0.039) and inversely with QOL (−11.5 [−17.8 to −5.25], *p* < 0.001) and SWLS (−2.19 [−4.3 to −0.10], *p* = 0.040) (Table 3).

**Figure 1:**
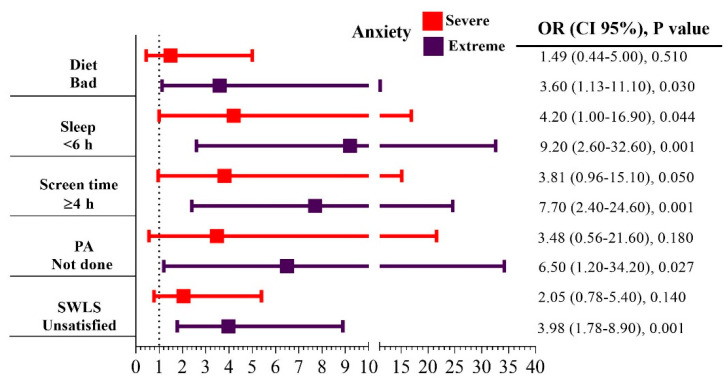

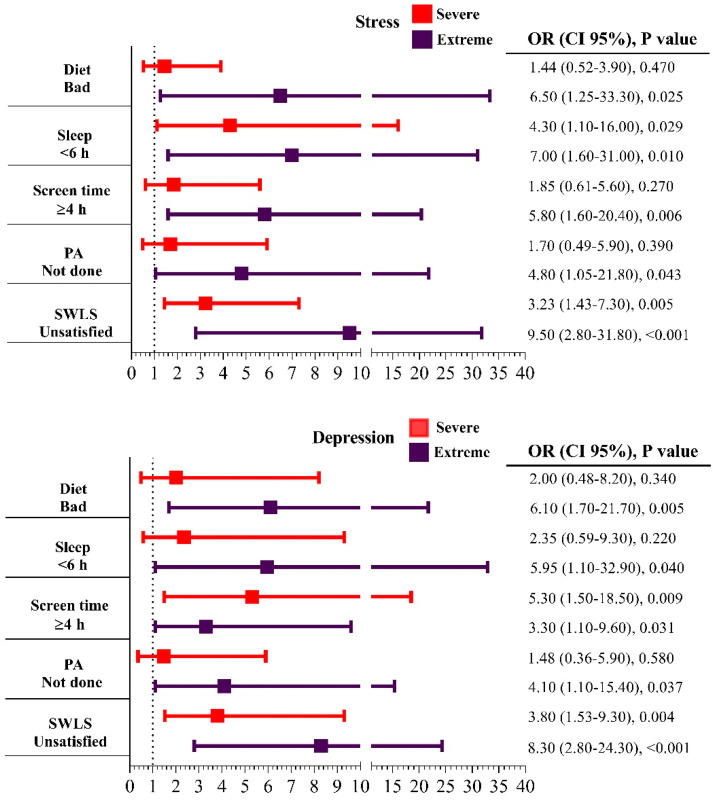
Associations between lifestyle and mental health. The figure shows the association between different lifestyle factors and the severity of anxiety, stress and depression.

Bad food habits (odds ratio [OR] = 3.60 [1.13–11.10], *p* = 0.030), <6 h of sleep (OR = 9.20, *p* = 0.001), ST (OR = 7.70, *p* = 0.001), physical inactivity (OR = 6.50, *p* = 0.027) and SWLS (OR = 3.98, *p* = 0.001) were associated with extreme anxiety. Similarly, bad diet (OR = 6.50, *p* = 0.025), < 6 h of sleep (OR = 7.00, *p* = 0.010), ST (OR = 5.80, *p* = 0.006), physical inactivity (OR = 4.80, *p* = 0.043) and SWLS (OR = 9.50, *p* < 0.001) were associated with severe stress. Finally, bad food habits (OR = 6.10, *p* = 0.005), <6 h of sleep (OR = 5.95, *p* = 0.040), ST (OR = 3.30, *p* = 0.031) and SWLS (OR = 8.30, *p* < 0.001) were associated with severe depressive symptoms (Figure 1).

## 4. Discussion

The objective of the present study was to investigate the association between mental health (i.e., anxiety, depressive symptoms and stress), QOL, SWLS and lifestyle parameters (i.e., PA, sleep duration, ST and food habits) among Chilean university students and then to determine the differences in mental health, QOL, SWLS and lifestyle parameters according to gender. The main results of this study were as follows: (i) the scores related to the DASS-21 were significantly higher in women; (ii) women mostly had a poor quality diet, whereas men had more ST hours per day but also more PA hours per week; (iii) in a model adjusted by age and gender, bad food habits (lowest score in the food survey) were related to anxiety, depressive symptoms and stress (iv) <6 h of sleep was related positively to poorer mental health, anxiety, depressive symptoms and stress, and inversely with QOL and SWLS; (v) ≥4 h of ST was linked positively to depressive symptoms and anxiety; and (vi) non-physical activity was related to anxiety, depression and stress scores and inversely with QOL and SWLS.

In the present study, the scores related to the DASS-21 were significantly higher in women. In this context, a longitudinal study indicated that anxiety was more prevalent in female university students and was the most serious issue [56]. Moreover, a cross-sectional study focused on university students reported that women tend to suffer more symptoms of stress, anxiety and depression than men [57]. Likewise, previous evidence suggests that gender was linked to the mental health profile [58]. Similarly, another study conducted in Mexican university subjects reported that female students had significantly higher scores for psychological distress and anxiety than male subjects [59]. Consistently with the above, previous evidence showed that female Chilean university students had a higher prevalence of anxiety, depression and stress [23]. There is evidence showing that women at university for three years presented with more anxiety and mental health problems compared with their counterparts [17]. Moreover, a study conducted in a representative sample indicated that being female seems to be the factor that has the greatest impact on anxiety and mental health problems in Chilean university students [16].

The above may be related to possible menstrual symptoms as a factor that increases levels of depression, anxiety and stress, from puberty to adulthood [60]. Moreover, we found that when categorizing the severity of negative emotions according to gender, women presented a greater proportion of severe and extreme anxiety. Additionally, data from university students have shown that severe to extreme anxiety was more prevalent in women (14.6%) than in men (8.1%) [61]. Building on previously reported findings, it is suggested that female university students are particularly vulnerable in terms of mental health [59]. Also, another study among university students showed that females had greater stress and anxiety than males [62]. On the other hand, another study indicated that mental health exhibited a non-significant difference by gender in medical students [63].

Women mostly had poor food habits, whereas men had more ST hours per day but also more PA hours per week. Consistently with the above, another study conducted in Spanish university students reported that the low prevalence of mental disorders was higher in women than in men [64]. In this regard, a cross-sectional study showed that men had better diet quality than women [65]. It also has been shown that dietary patterns differ according to gender [66]. In this sense, a previous study indicated that women had macronutrient imbalances, with a low consumption of fiber and a high intake of protein and fat [67]. Moreover, data from 210,106 subjects have shown that there were significant gender differences in adherence to dietary recommendations [68]. Furthermore, it has been suggested that men had more ST than women among university students [19]. Other evidence has suggested that males presented significantly higher ST than males in a representative sample of Norwegian university students [69]. Similarly, it has been demonstrated that ST is a risk factor for several negative health-related outcomes [70]. A previous investigation among university students showed that men had higher PA scores than women [71]. Consistently with the above, another study reported that male first-year university students were more physically active [72]. Similarly, another study conducted in university students reported that women presented a higher proportion of physical inactivity than men [73].

In a model adjusted by age and gender, bad food habits (lowest score in the food survey) were related to anxiety, depressive symptoms and stress. In this sense, another study conducted in university students showed that subjects with high stress showed less healthy dietary behaviors compared to subjects with low stress [74]. In addition, a cross-sectional study indicate that good food habits were linked with mental well-being in Italian university students [75].

Building on previously reported findings, it is suggested that during the university stage, unhealthy food patterns may be linked to poor mental health and health-related quality of life (HRQOL) [25]. Better diet quality seems to provide a buffer against mental health outcomes and depressive symptoms [23]. Other evidence suggested that healthy eating habits may contribute to better life satisfaction in Chilean university subjects [55]. It is also been shown that healthy eating habits were inversely related to depressive symptoms in youth [76]. Additionally, a university-population-based study indicated that Mediterranean diet adherence was positively linked with better HRQOL [77]. Other evidence has suggested that students with better diet quality had higher HRQOL, especially in the mental components [78]. Similarly, it has been shown that healthy eating habits were linked with better self-related general health in university students [79]. A cross-sectional study among university students showed that unhealthy eating habits and food insecurity were linked with high levels of depressive symptoms, anxiety and stress [80]. Moreover, a systematic review focusing on diet quality and mental health among university students showed that healthy eating habits were related to lower levels of depressive symptoms, stress and anxiety [81]. A recent study among university students reported that poor diet quality could have a negative effect on mental health [82]. Therefore, healthy life factors, such as eating habits, should be studied to create preventive interventions [75].

Furthermore, <6 h of sleep was related positively to poorer mental health, anxiety, depressive symptoms and stress, and inversely with QOL and SWLS. A cross-sectional study among university students showed that sleep quality could predict components of HRQOL [83]. Furthermore, it has been shown that baseline sleep quality outcomes were linked to depression, anxiety and stress, and therefore, a bidirectional relationship between sleep quality and mental health problems could exist in college students [84]. This relationship can be explained by the fact that poor sleep can exacerbate symptoms of anxiety and depression, while these symptoms can also lead to poorer sleep, creating a vicious cycle. Consistently with the above, a study among university students showed that poor lifestyle factors (i.e., less exercise and poor sleep quality) were linked to higher depressive symptoms [85]. This aligns with the understanding that physical activity can improve sleep quality and, consequently, mental health, emphasizing the interconnectivity of lifestyle factors. In this regard, meeting the sleep guidelines was strongly related to better mental health (i.e., depression and anxiety) [86]. Also, a previous study found that better sleep quality was associated with higher SWLS in university students, and therefore, SWLS could be used as an indicator of well-being [87].

Moreover, we found that ≥4 h of ST was linked positively to depressive symptoms and anxiety. In this sense, it has been shown that ST predicted higher depression, anxiety and stress among college students [35]. Another study among university students found that ST was linked with depression and anxiety [88]. Furthermore, it has been suggested that an unhealthy lifestyle may increase the risk of mental health problems [89]. Another study among university students from 24 countries reported that higher ST was linked with poorer SWLS [90]. Similarly, it has been reported that sleep duration is associated with SWLS [87]. In addition, non-physical activity was related to anxiety, depression and stress scores, and inversely related with QOL and SWLS. In this context, another study indicated that PA levels were positively linked to better mental health in university students [28]. Complementarily to the above, a study reported data from university students showing that PA was inversely related to anxiety and depressive symptoms [29]. In addition, another study among college students reported that SWLS was regulated positively by PA and negatively by ST; therefore, it is important to generate strategies that increase PA and reduce ST [91]. At the level of educational policies, in higher education, law No. 21.091 establishes the explicit right to educational and comprehensive training of individuals. In this context, the Ministry of Education, through the Under-Secretariat of Higher Education, has taken on the task of building a strategy for the promotion and prevention of mental health in Higher Education, revealing the importance of studying and contributing to this issue that responds to a better development of students’ educational trajectories.

### Limitations

One of the limitations of this study is its cross-sectional design, which precludes establishing definitive causal relationships between lifestyle factors and mental health outcomes. Another limitation is the assessment of lifestyle factors using self-report questionnaires, which could lead to recall or perception biases. In addition, some aspects of lifestyle, such as diet quality, may be subjective and vary according to the individual interpretation of the participants. Another limitation is not having considered some important variables such as BMI and socioeconomic status (SES); we plan to incorporate them in future studies.

Another limitation is the sample size of university students, which decreases the robustness of the results. Finally, although the sample size was calculated with a 95% confidence level and was selected purposively and non-probabilistically, its size is relatively small and concentrated on one faculty and three degree programs which, being specific, may restrict the generalizability of the findings to a wider student population. Likewise, it is important to highlight that the conclusions must consider that the results cannot be extrapolated because it is a pilot project with a low sample size.

## 5. Conclusions

In this study, a clear disparity in mental health components (including anxiety, depressive symptoms and stress) and QOL scores was found between male and female university students. The women showed significantly higher scores on the DASS-21, especially for anxiety, whereas the men had better physical health but showed higher ST and poorer diet quality. Moreover, this study showed that unhealthy lifestyle factors (i.e., insufficient sleep, lack of PA and prolonged ST) were strongly associated with poorer mental health and QOL in university students. The findings highlight the importance of addressing these aspects of lifestyle in intervention and health promotion programs aimed at young university students in order to improve their mental health and overall QOL.

## Figures and Tables

**Table 1:** Participants’ characteristics in mental health according to sex in the population.

Characteristics	Total *n* = 211	Men *n* = 83	Women *n* = 128	*p*-Value
Age (years old) ^a^	21 (18–28)	21 (18–29)	21 (18–26)	0.019
Negative emotional states				
DASS 21 score total ^a^	24 (4–56)	19 (4–52)	26 (5–56)	<0.001
Anxiety score ^a^	7 (0–20)	6 (0–19)	8 (1–20)	<0.001
Depression score ^a^	7 (0–20)	6 (0–19)	11 (3–19)	0.013
Stress score ^a^	10 (2–19)	9 (2–20)	11 (3–19)	0.002
Category anxiety, *n* (%) ^b^				0.004
Not present	62 (29.38)	36 (43.37)	26 (20.31)	
Light	19 (9.0)	8 (9.64)	11 (8.59)	
Moderate	33 (15.64)	12 (14.46)	21 (16.41)	
Severe	29 (13.74)	10 (12.05)	19 (14.84)	
Extreme	68 (32.23)	17 (20.48)	51 (39.84)	
Category depression, *n* (%) ^b^				0.184
Not present	71 (33.65)	36 (43.37)	35 (27.34)	
Light	33 (15.64)	11 (13.25)	22 (17.19)	
Moderate	42 (19.91)	14 (16.87)	28 (21.88)	
Severe	23 (10.9)	9 (10.84)	14 (10.94)	
Extreme	42 (19.91)	13 (15.66)	29 (22.66)	
Category stress, *n* (%) ^b^				0.076
Not present	71 (33.65)	35 (42.17)	36 (28.13)	
Light	21 (9.95)	9 (10.84)	12 (9.38)	
Moderate	44 (20.85)	19 (22.89)	25 (19.53)	
Severe	51 (24.17)	13 (15.66)	38 (29.69)	
Extreme	24 (11.37)	7 (8.43)	17 (13.28)	
HRQOL				
QOL Physical score	26 (18–32)	27 (18–32)	25 (17–32)	0.007
QOL Psychological score	20 (11–27)	22 (11–27)	19 (11–26)	0.013
QOL Social score	11 (6–15)	11 (5–15)	11 (6–15)	0.501
QOL Environmental score	28 (20–35)	29 (20–35)	27 (20–35)	0.269
QOL RAW score	84 (58–100)	89 (58–100)	81 (58–99)	0.022
Satisfaction with Life score	15 88–23)	16 (9–23)	15 (8–23)	0.223
Satisfaction with Life, *n* (%)				0.576
Extremely satisfied	35 (16.59)	16 (19.28)	19 (14.84)	
Satisfied	65 (30.81)	27 (32.53)	38 (29.69)	
Slightly satisfied	19 (9.0)	9 (10.84)	10 (7.81)	
Dissatisfied	72 (34.12)	23 (27.71)	49 (38.28)	
Extremely dissatisfied	20 (9.48)	8 (9.64)	12 (9.38)	

Abbreviations: DASS21; Depression Anxiety Stress Scale, QOL; Quality of Life. ^a^ Data are expressed as the median and percentiles 5th–95th, compared using Wilcoxon rank-sum test. ^b^ Data are expressed as the *n* (%), compared using the Chi-square test. *p*-value < 0.05 was considered statistically significant.

**Table 2:** Lifestyle parameters according to sex.

Characteristics	Total *n* = 211	Men *n* = 83	Women *n* = 128	*p*-Value
Food habits score	7 (3–12)	8 (2–12)	6 (3–12)	0.020
Food habits quality				0.014
Good	49 (23.2)	28 (33.7)	21 (16.4)	
Regular	96 (45.5)	33 (39.8)	63 (49.2)	
Bad	66 (31.3)	22 (26.5)	44 (34.4)	
Hours of sleep h/d	7 (5–9)	7 (5–8.5)	7 (5–9)	0.731
Sleep category				0.180
≥8 h	64 (30.3)	20 (24.1)	44 (34.4)	
6–7 h	111 (52.6)	50 (60.2)	61 (47.6)	
<6 h	36 (17.1)	13 (15.7)	23 (18.0)	
Screen time, h/d	2 (2–4)	3 (2–4)	2 (2–4)	0.004
Screen time category, *n* (%)				0.012
≤2 h	107 (50.7)	34 (41.0)	73 (57.0)	
3 h	63 (29.9)	25 (30.1)	38 (29.7)	
≥4 h	41 (19.4)	24 (28.9)	17 (13.3)	
Physical activity (PA) h/d	2 (0–5)	2 (0.5–6)	1 (0–4)	0.004
Category PA				0.006
Not done: 0 min	31 (15.2)	3 (3.8)	28 (22.4)	
≤60 min	60 (29.4)	24 (30.4)	36 (28.8)	
60–120 min	48 (23.5)	21 (26.6)	27 (21.6)	
121–180 min	32 (15.7)	17 (21.5)	15 (12.0)	
>180 min	33 (16.2)	14 (17.7)	19 (15.2)	

Abbreviations: DASS21; Depression Anxiety Stress Scale, QOL; Quality of Life.

**Table 3:** Effect of lifestyle factors on mental health parameters.

Characteristics	ß (CI 95%)	ß (CI 95%)	ß (CI 95%)	ß (CI 95%)
Negative Emotional States	Bad Food Habits	<6 h of Sleep	≥4 h Screen Time	Non-Physical Activity
DASS 21 score total	8.29 (2.7–13.9), 0.004	13.5 (7.6–19.5), 0.001	8.3 (2.86–13.7), 0.003	7.7 (1.0–14.3), 0.240
Anxiety score	2.3 (0.22–4.36), 0.03	4.2 (2.0–6.4), <0.001	3.45 (1.47–5.4), 0.001	2.6 (0.20–5.0), 0.030
Depression score	3.75 (1.54–5.9), 0.001	5.5 (3.2–7.9), <0.001	3.2 (1.05–5.4), 0.004	2.7 (0.009–5.3), 0.049
Stress score	2.24 (0.31–4.17), 0.023	3.8 (1.8–5.9), <0.001	1.63 (−0.23–3.5), 0.087	2.4 (0.12–4.7), 0.039
HRQOL				
QOL Physical score	−3.8 (−5.53 to −2.0), <0.001	−4.35 (−6.2 to −2.5), <0.001	−2.13 (−3.88 to −0.37), 0.018	−3.56 (−5.6 to −1.47), 0.001
QOL Psychological score	−3.97 (−5.7 to −2.23), <0.001	−4.32 (−6.2 to −2.4), <0.001	−1.77 (−3.55 to −0.005), 0.05	−3.55 (−5.6 to −1.48), 0.001
QOL Social score	−0.88 (−1.91 to 0.15), 0.095	−2.1 (−3.2 to −1.0), <0.001	0.54 (−0.46 to 1.55), 0.290	−1.76 (−2.96 to −0.55), 0.004
QOL Environmental score	−3.35 (−5.2 to −1.53), <0.001	−3.0 (−5.0 to −1.0), 0.003	−0.69 (−2.52 to 1.13), 0.457	−2.63 (−4.81 to −0.44), 0.019
QOL RAW score	−12.0 (−17.2 to −6.8), <0.001	−13.8 (−19.4 to −8.2), <0.001	−4.05 (−9.4 to 1.27), 0.135	−11.5 (−17.8 to −5.25), <0.001
Satisfaction with Life score	−3.77 (−5.5 to −2.06), <0.001	−4.27 (−6.1 to −2.4), <0.001	−2.15 (−3.86 to −0.43), 0.015	−2.19 (−4.3 to −0.10), 0.040

Abbreviations: DASS21; Depression Anxiety Stress Scale, QOL; Quality of Life. Data are expressed as the median and percentiles 5th–95th, compared using Wilcoxon rank-sum test. Data are expressed as the n (%), compared using the Chi-square test. *p*-value < 0.05 was considered statistically significant.

## Data Availability

The original contributions presented in the study are included in the article, further inquiries can be directed to the corresponding author.
